# University of Nashville

**Published:** 1857-03

**Authors:** 


### UNIVERSITY OF NASHVILLE.

We have received, and read with pleasure, mingled with regret, the address to the class of the University of Nashville, at the commencement of the present session, delivered by Prof. Lindsley. With pleasure, because it is a merited and interesting tribute to the memory of a noble member of our profession; and, with regret, that he should so soon have passed away.

Though, we had not the pleasure of a personal acquaintance with Prof. Porter, from all that we have been able to learn of him, we have no doubt the University of Nashville has sustained a serious loss in his death.

We understand that the present class in that institution, is a much larger one than the last, and with a continuance of increasing classes, it must, in a very short time, become the leading school (so far, at least, as the number of students in attendance, can make it so,) in the United States.

To the high destiny which seems to await the University of Nashville, we have no objection; it is an institution, taken altogether, indeed, to which we look with pride and pleasure, more particularly as the result of Southern energy and talent, and as a demonstration of the fact, that Southern institutions can be made successful. It is very true—as connected with another enterprise, on account of what we consider an illiberal and ungenerous course, unworthy of those who should control the halls of scicnce and learning—we *owe* them no good will; and if we were disposed to be revengeful, (taking into consideration the arrogant and selfish spirit, which has been manifested towards the Atlanta Medical College,) we might remark, that "Pride cometh before a fall;" we prefer, however, to indulge a different mood, and to predict good rather than evil; to which, however, we must confess we are prompted in a good degree, by the feeling, that we have attained a position of security, and independence of their efforts to injure us, by which we are enabled to *afford* a forgiving spirit.

